# Tissue Doppler Imaging and strain rate of the left atrial lateral wall: age related variations and comparison with parameters of diastolic function

**DOI:** 10.1186/s12947-020-00221-2

**Published:** 2020-09-10

**Authors:** Laura V. Argento, Carolina M. Travetto, Maria de las M. Colicigno, Gerardo Marambio, Silvia Gentile, Ana Salvati, Jorge Lax, Tomás Cianciulli

**Affiliations:** 1 Hospital General de Agudos Dr. Cosme Argerich, Pi y Margall 750 (C1155AHD) CABA, Buenos Aires, Argentina; 2 Sanatorio Clínica Modelo de Modelo de Morón, Buenos Aires, Argentina; 3 Fellow of the American College of Cardiology, Washington DC, USA; 4 Fellow of the American Society of Echocardiography, Durham, USA

**Keywords:** Diastolic function; tissue Doppler echocardiography, Strain rate imaging, Left atrium

## Abstract

**Background:**

Strain Rate Imaging (SRI) is one of the most used techniques to study left atrial (LA) and diastolic function. Its availability in low-income countries is diminished since it requires additional expensive software, among other limitations. In contrast, Tissue Doppler Imaging (TDI) is widely available and easy to use. We hypothesize TDI could detect changes in LA and diastolic function associated with age similarly to SRI. The aim of this study is to evaluate the effects of age on LA and diastolic function assessed by LA lateral wall TDI online by spectral pulse, and to compare them with age-related variations of LA SRI and other parameters of diastolic function in a population of healthy adults.

**Materials and methods:**

Ninety-one healthy adults were prospectively evaluated. In apical four - chamber view the LA lateral wall was divided in three portions. Peak velocities of basal and mid portions were measured with TDI online by spectral pulse and with SRI by speckle tracking. A first positive wave (*S’la* and SRS) and two negative waves (*E’la* and SRE, and *A’la* and SRA respectively) were obtained. E’la/A’la ratio and SRE/SRA ratio were analyzed. The distribution of the variables by age subgroups was described and analyzed. Correlation analyses were performed.

**Results:**

The median age was 42 years old and 54.9% were female. *E’la/A’la* showed a negative good correlation with age. *E’la/A’la* and SRE/SRA ratios changed from > 1 to < 1 in the age group of 41–50 years old, while this occurred in the group of 51–60 years old for the E/A ratio. Lateral and septal mitral annulus E´ showed decrease with age and prolongation of E-wave deceleration time was observed in the age group over 61 years old.

**Conclusion:**

Normal values​​ according to age group of TDI of the LA lateral wall were obtained. Age-related changes in LA and diastolic function could be detected as early with TDI as with SRI. Future studies are required to explore if this method could be used to address in part LA or diastolic function in other populations with established cardiovascular disease or at risk of presenting it, which could be useful in low-income settings, where SRI is not available.

## Background

With senescence, a series of changes occur at the myocyte level, which results in a reduction of left ventricular (LV) distensibility and, consequently, at the atrial level in a decrease in the conduit phase and a compensatory increase of LV filling during atrial systole [1–4].

Several studies have shown an association between atrial and diastolic dysfunction and the development of cardiovascular events during follow up, particularly atrial fibrillation (AF) [5–8] and heart failure in patients with preserved LV systolic function [9–11].

The evaluation of the left atrium (LA) by Echo Doppler is in expansion; new techniques have been developed in recent years, including Tissue Doppler Imaging (TDI) and Strain Rate Imaging (SRI). Impaired atrial function detected by SRI has been associated with adverse cardiovascular events [12, 13].

The evaluation of atrial and diastolic function by SRI can be a challenge in low-income countries or centers since it requires using additional expensive software, among other limitations. On the other hand, TDI is a simple technique and is currently widely available.

Previously, we have reported the associations observed between the velocity profile of LA lateral wall measured by TDI online by spectral pulse with atrial SRI by speckle tracking, and other parameters of atrial and diastolic function, in a population of adults without known cardiovascular disease [14]. This report consists of a sub-analysis of that study and seeks to describe the age-related variations observed in the parameters analyzed in a normal population, in order to establish an initial reference value necessary, as a previous instance, for the study of atrial and diastolic function through this method in other populations with established cardiovascular pathology or at risk of presenting it.

## Materials and methods

The aim of this study is to evaluate the effects of age on LA and diastolic function assessed by LA TDI online by spectral pulse, and to compare them with age-related variations of LA SRI and parameters of diastolic function in a population of healthy adults.

As previously reported, 91 healthy adults, aged between 18 and 74 years old, were prospectively evaluated in two health centers between April 2016 and March 2018. None of the subjects had a known history of cardiovascular disease, high blood pressure, dyslipidemia, diabetes, electrocardiographic alterations or was under cardiovascular medical treatment. A complete color Doppler echocardiogram was performed.

### Echocardiogram and cardiac Doppler

An ultrasound machine Vivid S5 or 7 (GE Medical System, Horten, Norway), equipped with a 3 MHz variable frequency transducer, was used for all of the echocardiographic evaluations. Cardiac diameters were measured according to the recommendations of the ASE [15]. The M mode was used to measure the diameters of LA and LV. Volumes of LA were measured in a single-plane apical four-chamber view, using the method of discs.

Transmitral flow velocities were obtained by pulsed wave Doppler echocardiography, in an apical four-chamber view. Mitral flow parameters measured included peak velocities during early diastole (E) and late diastole (A), their ratio (E/A ratio), and E-wave deceleration time (DTE).

TDI online by spectral pulse of the septal and lateral mitral annulus was performed, and peak of the systolic wave S′ (Sma S´, Lma S´), early filling wave E’ (Sma E´, Lma E´) and late filling wave A’ (Sma A´, Lma A´) were measured. The E/e’ average ratio was calculated.

Parameters used to define normal diastolic function were: Lma E’ > 10 cm/s, Sma E’ > 7 cm/s, average E/e’ < 14, peak velocity of tricuspid regurgitation < 2.8 m/s, LA volume indexed < 34 ml/m^2^ [16].

**Fig. 1:**
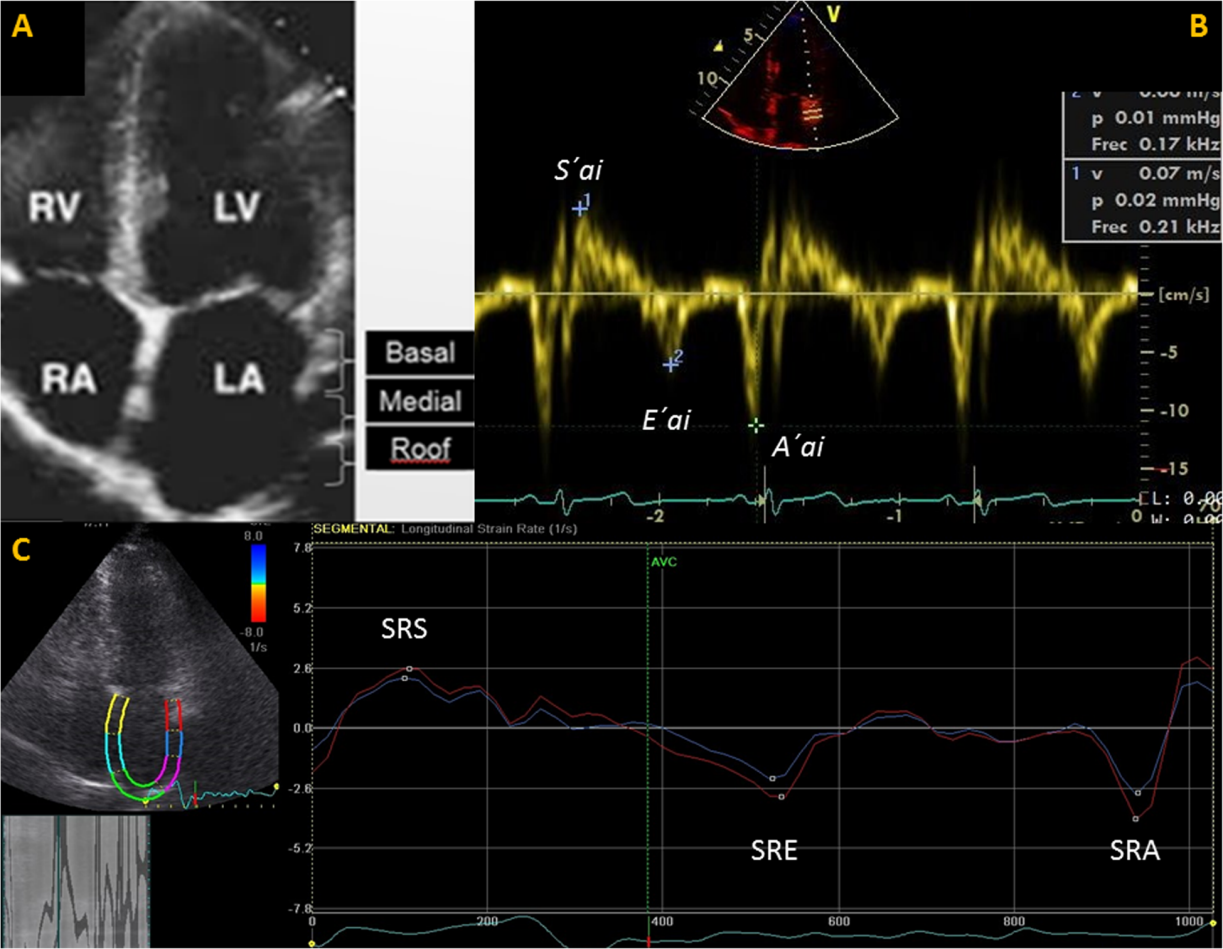
Methodology used for the evaluation of LA lateral wall ​​by TDI and SRI. A) Subjective division of the LA lateral wall in three segments; B) TDI on line by spectral pulse at basal segment of LA lateral wall. Three waves are identified: *S’la* (evaluates reservoir function), *E’la* (evaluates conduit function), *A’la* (evaluates pump function). C) LA SRI by speckle tracking with the analysis of the basal and mid segments. Three waves are identified: SRS (evaluates reservoir function); SRE (evaluates conduit function) and SRA (evaluates pump function). For more information, refer to the text. LA: left atrial, TDI: Tissue Doppler Image, SRI: Strain Rate Image, *la*: left atrial

For the analysis of LA function by TDI and SRI, lateral wall was obtained in the apical four chamber view and divided subjectively into three portions: basal segment, mid segment and atrial roof (Fig. 1a). TDI by spectral pulse measures were taken online, in apnea, at the end of expiration, with a sample volume of 2 mm and trying to maintain an angle less than 15°; gain and filter were adjusted to avoid saturation of the image. Three consecutive measurements of basal and mid segments were performed and the best spectral image of each segment was selected for the analysis; and peak velocities of this waves were measured, the atrial roof was excluded from the measurements.

The Echo Pac software v 108.1.5 (GE Healthcare) was used for the analysis of two-dimensional SRI by speckle tracking. In an apical four-chamber view, the endocardial surface of the LA was manually drawn, using a single cardiac cycle and the R wave as a reference point. A region of interest was automatically generated, and manually tracked, frame by frame, to maintain its position within the LA wall, to ensure that the regions of interest would follow the cardiac movement throughout cardiac cycle. The LA ​​was divided into six segments automatically; the limits of the basal and mid segments of the lateral wall of the LA were manually adjusted to resemble the division made by TDI, excluding the atrial roof and the remaining segments. All the images were obtained at an average frame rate of 63 +/− 6.

With both techniques, TDI and SRI, three waves were obtained: a first positive wave, *S’la* and SRS (reservoir phase), and two negative waves, *E’la* and SRE (conduit phase) and *A’la* and SRA (atrial systole) respectively (Fig. 1b and c). The ratios between *E’la/A’la* and SRE/SRA were analyzed. Basal and mid segments were analyzed, as well as the average between basal and mid segments when both curves could be measured. Segments that did not have adequate image quality were excluded from the analysis. Likewise other authors [17], we did not include the interatrial septum since it is very thin and largely made up of collagen tissue.

### Repeatability and reproducibility

In 30 randomly selected subjects TDI measurements were performed independently and blindly by two operators, with a difference of 10 min between studies. Repeatability was evaluated by comparing the first two measurements performed by the first operator; reproducibility was evaluated by comparing the first measurement of each of the operators. Although the average of the multiple measures performed by each operator would inform lower values ​​of variability, comparison of a single measurement was chosen for external validity reasons, given that in clinical practice the operator would not be expected to make multiple measurements and average them for report.

#### Statistical analysis

Absolute frequency and percentage were used as frequency measures for qualitative variables; median (Md) and interquartile range (IQR) were used as frequency and dispersion measures for quantitative variables, given that the variables under study did not present normal distribution. We analyzed the distribution of the variables in the total population and by subgroups of age (< 30, 31–40, 41–50, 51–60 and ≥ 61 years). The comparisons between subgroups of age were performed with the Kruskal-Wallis method. Considering age as a continuous variable, correlation analyzes were performed to evaluate the existence of linear association between age and various parameters, using Spearman’s correlation method with Holm’s method adjustment. After log transformation of the abnormally distributed variables, univariable and multivariable linear regression analyzes were performed to assess association between TDI parameters and clinical predictors. Variables associated in univariate analyses were entered in multivariable models. Intra and inter-observer variabilities were assessed using the Bland-Altman method, and presented as mean difference (MD), standard deviation (SD) and coefficient of repeatability (COR), calculated as 2 x SD of the differences in repeated measurements [18]. In the hypothesis tests a statistical significance level of 0.05 was used, rejecting the null hypothesis if the *p*-value was lower than this level. All data analysis was performed using R Core Team 2018 software (R: A language and environment for statistical computing, R Foundation for Statistical Computing, Vienna, Austria. URL https://www.R-project.org/).

## Results

### Clinical characteristics and echocardiographic measures of the study population according to age subgroups

The Md age was 42 years old (IQR 18) and 54.9% of the subjects were female. An adequate spectral signal was obtained in 99 and 92% of the basal and mid segments with TDI and in 90 and 84% with SRI, respectively. Table 1 shows the global values and the distribution of the variables according to age subgroups (data of TDI and SRI correspond to the basal segments; the values ​​of mid segments and average between of basal and mid segments are presented in Supplemental Table 1). The older age subgroups presented higher values ​​of systolic and diastolic blood pressure (SBP and DBP). No differences were observed between the subgroups in other clinical variables evaluated. Of the diastolic function parameters evaluated, progressive reduction of E wave values and parallel increase in A wave values ​​were observed at increasing age, with reversal of the E/A ratio in the subgroups older than 50 years old. Age-related DTE prolongation > 240 ms, reduction of Sma E´ and Lma E´ waves, and increase of Sma A´ and Lma A´ waves were also observed. Sma E´/Sma A´ turned < 1 in age subgroups over 50 years old and Lma E´/Lma A´ turned < 1 in age subgroups over 60 years old. Sma E´ and Lma E´ presented values ​​below 10 cm/s and 7 cm/s, respectively, in the subgroups over 60 years old. The E/e´ ratio increased with age, with the Md in all groups being less than 10.

**Table 1:** Global parameters and analysis of the variables by age subgroups

	**Global** **Md (IQR)**	**≤ 30 y** **n: 14** **Md (IQR)**	**31–40 y** **n: 27** **Md (IQR)**	**41–50 y** **n: 27** **Md (IQR)**	**51–60 y** **n:14** **Md (IQR)**	**≥ 61 y** **n: 9** **Md (IQR)**	***p***
**Age (y)**	42 (18)	23.5 (3.7)	34 (3.5)	44 (4)	53.5 (5.7)	69 (2)	< 0.0001
**Female (%)**	54.9	64.3	33.3	63	64.3	66.7	NS
**HR (bpm)**	74 (15)	76.5 (11.7)	74 (15)	75 (14.2)	69.5 (9.7)	77 (12)	NS
**SBP (mmHg)**	110.5 (16.5)	95 (18)	110 (19)	115 (16)	115 (17.5)	126 (8.7)	< 0.0001
**DBP (mmHg)**	70.5 (10)	60 (10)	75 (10)	76 (10)	80 (10)	77.5 (10)	0.001
**BSA**	1.83 (0.2)	1.71 (0.1)	1.97 (0.3)	1.83 (0.19)	1.84 (0.4)	1.76 (0.1)	0.03
**EF (%)**	63 (11.7)	61 (8)	63 (13.5)	66 (11)	62 (7)	62.5 (12.2)	NS
**LA Vol Ind (ml/m2)**	22.9 (8)	20.6 (6.3)	21.7 (5.4)	23 (12.4)	25.2 (5.5)	22.8 (3.8)	NS
**E (cm/s)**	79 (22.5)	84.5 (15)	80 (23.5)	80 (17.5)	72.5 (20.5)	60 (11)	0.01
**A (cm/s)**	61 (23.5)	47.5 (15.2)	52 (14)	64 (20)	80 (17.7)	78 (20)	< 0.0001
**E/A**	1.32 (0.7)	1.79 (0.7)	1.62 (0.4)	1.22 (0.5)	0.98 (0.3)	0.7 (0.3)	< 0.0001
**DTE (ms)**	211 (70.5)	201.5 (68)	190 (44)	202 (63)	228.5 (45)	292 (152)	0.003
**LmaS´ (cm/s)**	10 (3)	11 (2.7)	11 (4.5)	11 (4)	9 (2.5)	10 (3)	NS
**LmaE´ (cm/s)**	14 (7)	17.5 (1.7)	16 (5)	14 (4.5)	11 (3)	9 (2)	< 0.0001
**LmaA´ (cm/s)**	9 (4)	6.5 (3.7)	8 (2.5)	9 (3.5)	9.5 (2.7)	13 (4)	0.001
**LmaE´/LmaA´**	1.70 (1.12)	2.70 (2.48)	2.0 (1.09)	1.37 (0.65)	1.10 (0.57)	0.6 (0.37)	< 0.0001
**SmaS´ (cm/s)**	8 (2)	7 (1)	8 (1)	8 (2)	7.5 (1)	8 (1)	NS
**Sma E´ (cm/s)**	9 (4)	11 (3.5)	11 (5)	9 (3)	8 (2.7)	6 (2)	0.001
**Sma A´ (cm/s)**	9 (3)	7 (2)	8 (3.5)	9 (3)	9 (1)	11 (3)	0.001
**Sma E´/Sma A´**	1.11 (0.81)	1.73 (0.49)	1.37 (0.92)	1 (0.39)	0.88 (0.31)	0.57 (0.18)	< 0.0001
**E/e´**	6.7 (1.7)	5.77 (1.3)	6.17 (1)	7.01 (1.5)	7.68 (2.4)	8.4 (1.7)	0.0002
***S’la*** **(cm/s) basal**	11.5 (3)	10 (2)	11 (2)	12 (3)	11 (3.7)	12 (3)	NS
***E’la*** **(cm/s) basal**	13 (5)	15 (5)	16 (3)	13 (3)	10 (2)	9 (1)	< 0.0001
***A’la*** **(cm/s) basal**	12 (4)	10 (3)	11 (2)	14 (5)	12.5 (2.7)	13 (2)	0.0006
***E’la/A’la basal***	1.06 (0.6)	1.63 (0.5)	1.42 (0.4)	0.92 (0.2)	0.82 (0.2)	0.64 (0.1)	< 0.0001
**SRS (1/s) basal**	2.9 (0.9)	3.6 (1.2)	3.13 (0.8)	2.62 (1.1)	2.54 (0.8)	2.17 (1.1)	0.016
**SRE (1/s) basal**	- 2.74 (1.1)	−3.4 (1)	−3.06 (0.6)	−2.26 (1.2)	−2.31 (0.5)	−1.97 (0.2)	< 0.0001
**SRA (1/s) basal**	−2.65 (1.1)	−2.6 (1.2)	−2.48 (1.1)	−2.7 (1.1)	− 2.9 (0.8)	−2.87 (0.7)	NS
**SRE/SRA basal**	1.03 (0.6)	1.23 (0.1)	1.19 (0.5)	0.78 (0.6)	0.88 (0.2)	0.69 (0.1)	0.002

*HR* heart rate, *bpm* beats per minute, *SBP* systolic blood pressure, *DBP* diastolic blood pressure, *BSA* body surface area, *EF* ejection fraction, *LA Vol Ind* indexed left atrial volume, *DTE* E-wave deceleration time, *Lma* lateral mitral annulus, *Sma* septal mitral annulus, *la* left atrial, *SR* Strain Rate

**Fig. 2:**
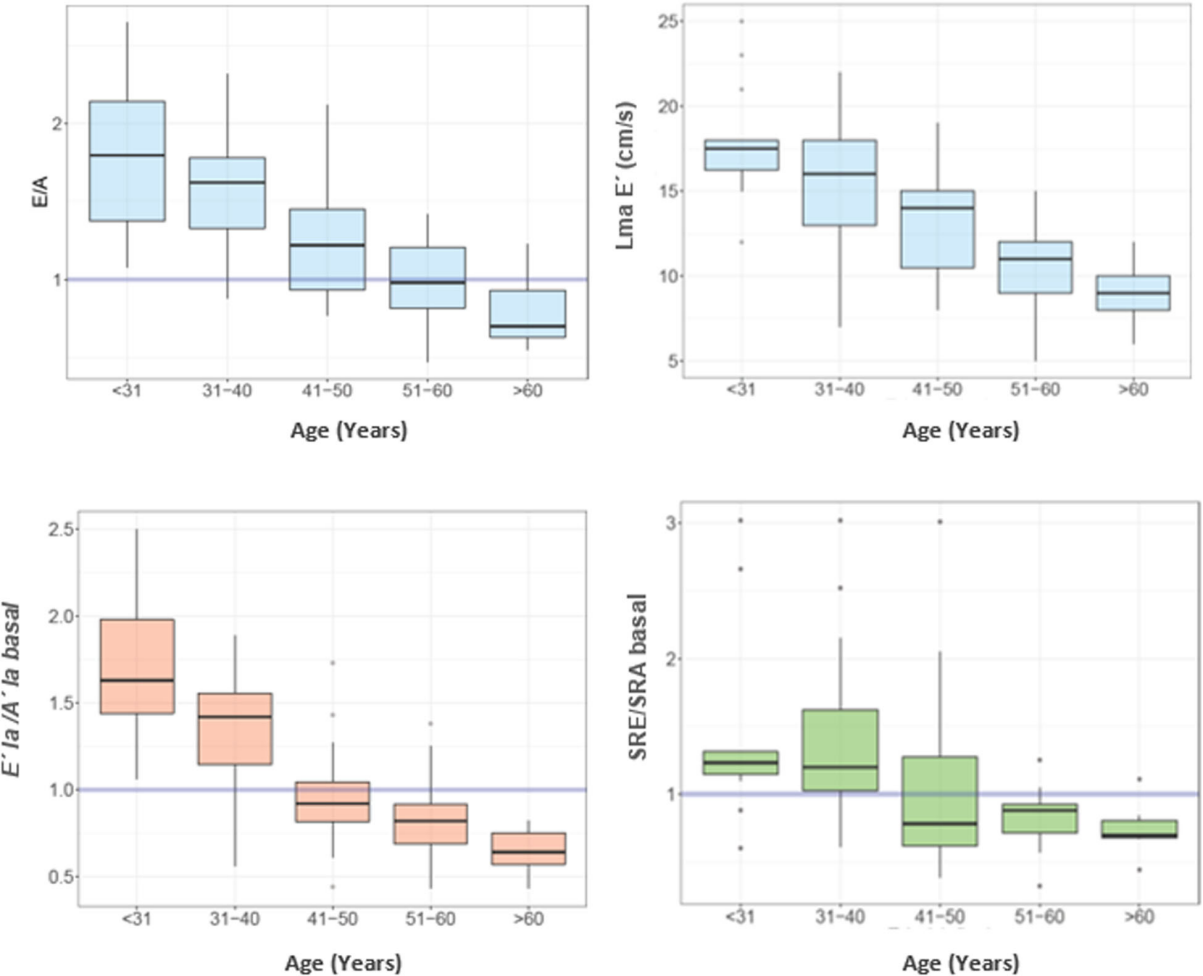
Box plot: analysis of age in relation to the variable E/A, LmaE´ basal, *E’la/A’la* basal, SRE/SRA. Lma: left mitral annulus, *la*: left atrial, SR: Strain Rate

No age related changes were observed in *S’la* wave at the basal segments, unlike that observed with SRS, which presented lower values at increasing age. Both *E’la* and SRE presented progressive variations with age, with a reduction in *E’la* values ​​and an increase in SRE values ​​in the older age subgroups. *A’la* wave values presented significant differences between the age subgroups, and no significant differences were observed between the groups in the distribution of SRA. *E’la/A’la* and SRE/SRA ratios < 1 were found in age subgroups over 40 years old (Table 1, Fig. 2). Similar findings were observed when analyzing mid segments and the average between basal and mid segments (Supplemental Table 1).

### Associations observed between age, clinical characteristics and echocardiographic measures

**Fig. 3:**
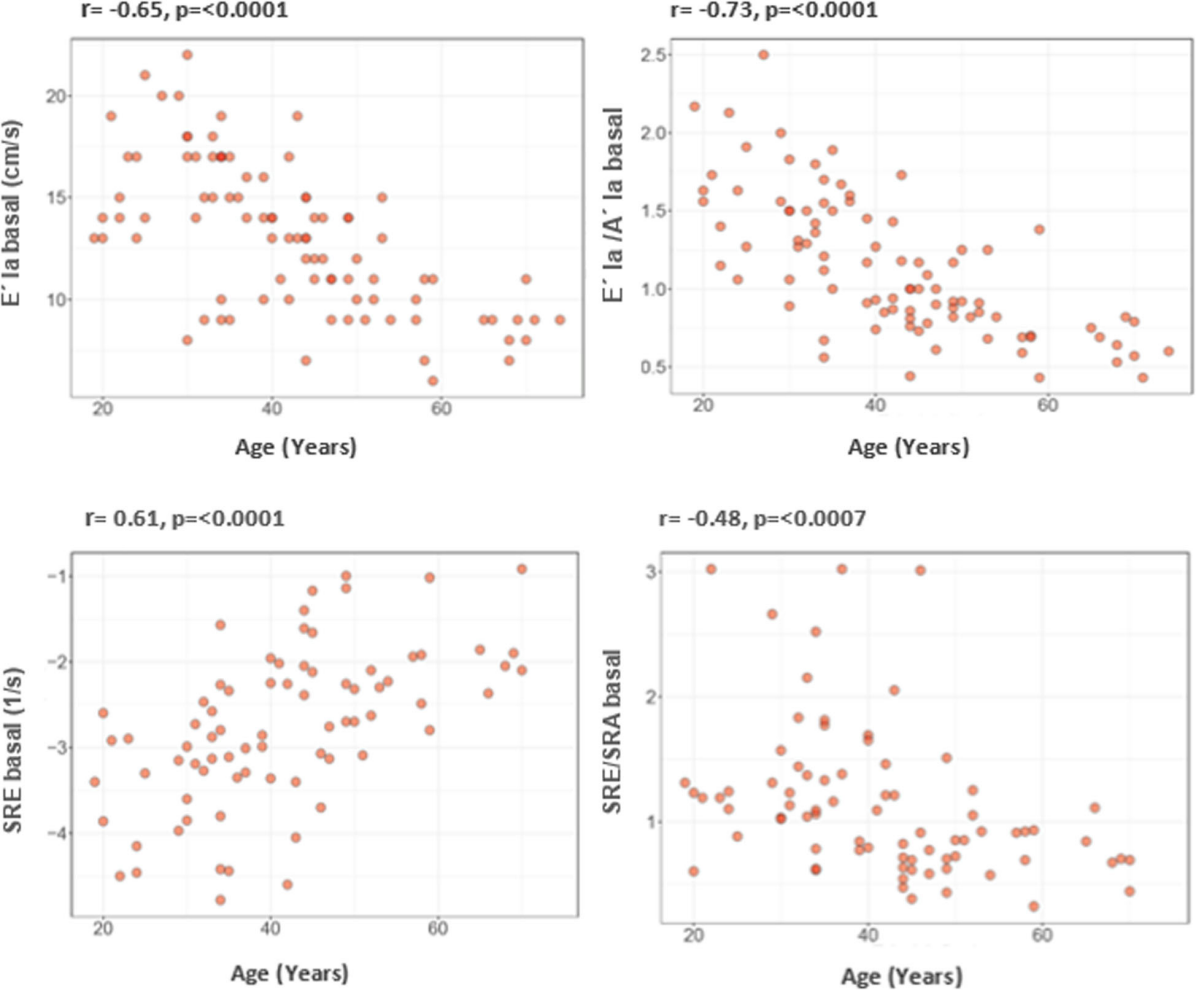
Linear correlations with age obtained in the conduit phase and the ratio between the conduit phase and atrial contraction using TDI and SRI. TDI: Tissue Doppler Imaging SR: Strain Rate

Table 2 and Fig. 3 present the results of the age correlations analyzed. SBP, A wave, E/e’ ratio and SRE basal presented a positive correlation with age, while Sma E´, Lma E´, E/A, Sma E´/Sma A´, Lma E´/Lma A´, *E’la*, SRS (mid and average), *E’la/A’la* and SRE/SRA at basal segments were negatively correlated; similar correlations with age were found at mid and average segments (Supplemental Table 2).

**Table 2:** Correlations with age

**Parameter**	***R***	***P***
LA Volume Indexed (ml/m2)	0.18	NS
SBP (mmHg)	0.44	0.04
DBP (mmHg)	0.35	NS
E (cm/s)	−0.39	NS
A (cm/s)	0.63	< 0.0001
E/A	−0.69	< 0.0001
DTE (ms)	0.31	NS
E/e´	0.47	0.002
Lma S´ (cm/s)	−0.26	NS
Lma E´ (cm/s)	−0.69	< 0.0001
Lma A´ (cm/s)	0.40	NS
Lma E´/Lma A´	−0,62	< 0.0001
Sma S´ (cm/s)	−0.12	NS
Sma E´ (cm/s)	−0.47	0.002
Sma A´ (cm/s)	0.34	NS
Sma E´/Sma A´	−0,53	< 0.0001
*S’la* (cm/s) basal	−0,01	NS
*E’la* (cm/s) basal	- 0.65	< 0.0001
*A’la* (cm/s) basal	0.36	NS
*E’la/A’la basal*	- 0.73	< 0.0001
SRS (1/s) basal	- 0.39	NS
SRE (1/s) basal	0.61	< 0.0001
SRA (1/s) basal	−0.06	NS
SRE/SRA basal	- 0.48	0.007

*LA* left atrial, *SBP* systolic blood pressure, *DBP* diastolic blood pressure, *DTE* E-wave deceleration time, *Lma* lateral mitral annulus, *Sma* septal mitral annulus, *la* left atrial, *SR* strain Rate

### Associations observed between TDI measures and clinical characteristics

Univariable and multivariable linear regression models for clinical predictors of LA TDI are summarized in Supplemental Table 3. Age was the most important independent predictor of *E’la* basal, *A’la* basal y *E’la/A’la* basal.

### Repeatability and reproducibility

Table 3 shows the intra-observer and inter-observer variabilities of LA TDI. Bland-Altman analysis showed no evidence of any systematic difference regarding intra or inter-observer variabilities. Greater variability was observed in the measurements of the mid segments, compared to the measurements of the basal segments.

**Table 3:** Intra-observer and inter-observer variabilities of LA TDI

Variable	INTRA-OBSERVER		INTER-OBSERVER	
	MD ± SD	COR	MD ± SD	COR
*S’la basal*	0.06 ± 1.01	2.02	0.56 ± 1.56	3.12
*E’la basal*	0.16 ± 1.01	2.02	−0.26 ± 1.85	3.7
*A’la basal*	0.03 ± 1.21	2.42	0.2 ± 1.91	3.82
*S’la mid*	0.16 ± 1.28	2.56	0.9 ± 1.9	3.8
*E’la mid*	0.06 ± 1.41	2.82	0.53 ± 2.52	5.04
*A’la mid*	0.1 ± 1.39	2.78	0.06 ± 2.21	4.42

*MD* mean difference, *SD* standard deviation, *COR* coefficient of repeatability*, la* left atrial

## Discussion

During the cardiac cycle the LA plays the functions of reservoir, conduit and atrial pump; the different phases are influenced by changes at the atrial chamber and the left ventricular chamber [2, 19].

Cell remodeling will occurs with normal aging, with loss of cardiomyocytes and compensatory development of hypertrophy; changes in the extracellular matrix were also described, with greater presence of fibrosis and deposits of amyloid protein [20]. At the molecular level, mitochondrial dysfunction and oxidative stress occur, as well as alterations in the entrance of calcium to the sarcoplasmic reticulum, which determine changes in cell elasticity with decreased relaxation and lower suction force by the LV, decrease in passive ventricular filling, and compensatory increase of the contribution of the atrial systole to LV filling to maintain an adequate stroke volume [21–23]. These events were associated with greater predisposition to suffer AF, and heart failure with preserved systolic function [24], entities that are becoming more prevalent in Argentina and the rest of the world [3, 25–28], given the increase in longevity that the world population is experiencing [2, 10].

New technologies in medicine are important components of health care, have the potential to save lives and improve quality of life and well-being. However, too many people around the world do not have access to affordable and high-quality health technology, and the problem is more serious in low and middle-income countries [29].

Different authors found an association between cardiovascular events and alteration in LA function evaluated by Strain and SRI. Inaba et al. found that SRS, SRE and SRA were lower in AF patients than in age-matched controls [30]. Wang et al. found that LA SRE and LA dimension were independent predictors of cardiovascular failure [31]. Mondillo et al. found that reservoir and conduit phases were lower in patients with hypertension or diabetes than in controls [32].

Many echo laboratories do not have the possibility of assessing LA by strain since it requires additional and expensive software, as well as a wide learning curve and post-processing of the images. Unlike SRI, TDI is a simple and widely available method that allows analyzing the information at the time of the study and does not require an extensive learning curve. This led us to think it could be used, to assess LA and diastolic function in low-resource centers where SRI is not available.

As a previous instance for the study of LA and diastolic function through this method in individuals with established cardiovascular pathology or at higher risk of presenting it, we need to understand how these parameters behave in a normal population and stablish initial reference values according to age groups, which is the reason why we perform this sub-analysis.

In this study we described the age-related changes observed in a population of adults without cardiovascular disease with TDI online, SRI and other diastolic function parameters. The variations in the velocities of *E’la* and SRE waves, and *E’la/A’la* and SRE/SRA ratios identified earlier the increase in the atrial systole contribution to LV filling than other diastolic function parameters.

### Comparison with previous studies

Pérez Paredes et al. [17] studied LA lateral wall with TDI by spectral pulsed online in mid segments, finding a decrease in the early diastole wave and an increase in late diastole wave in the age group over 45 years old; no changes in the reservoir phase were reported. Similar results were also obtained by Thomas et al. [33] when evaluating late diastole wave of the LA lateral wall at the annular and mid segments levels with color TDI offline.

**Fig. 4:**
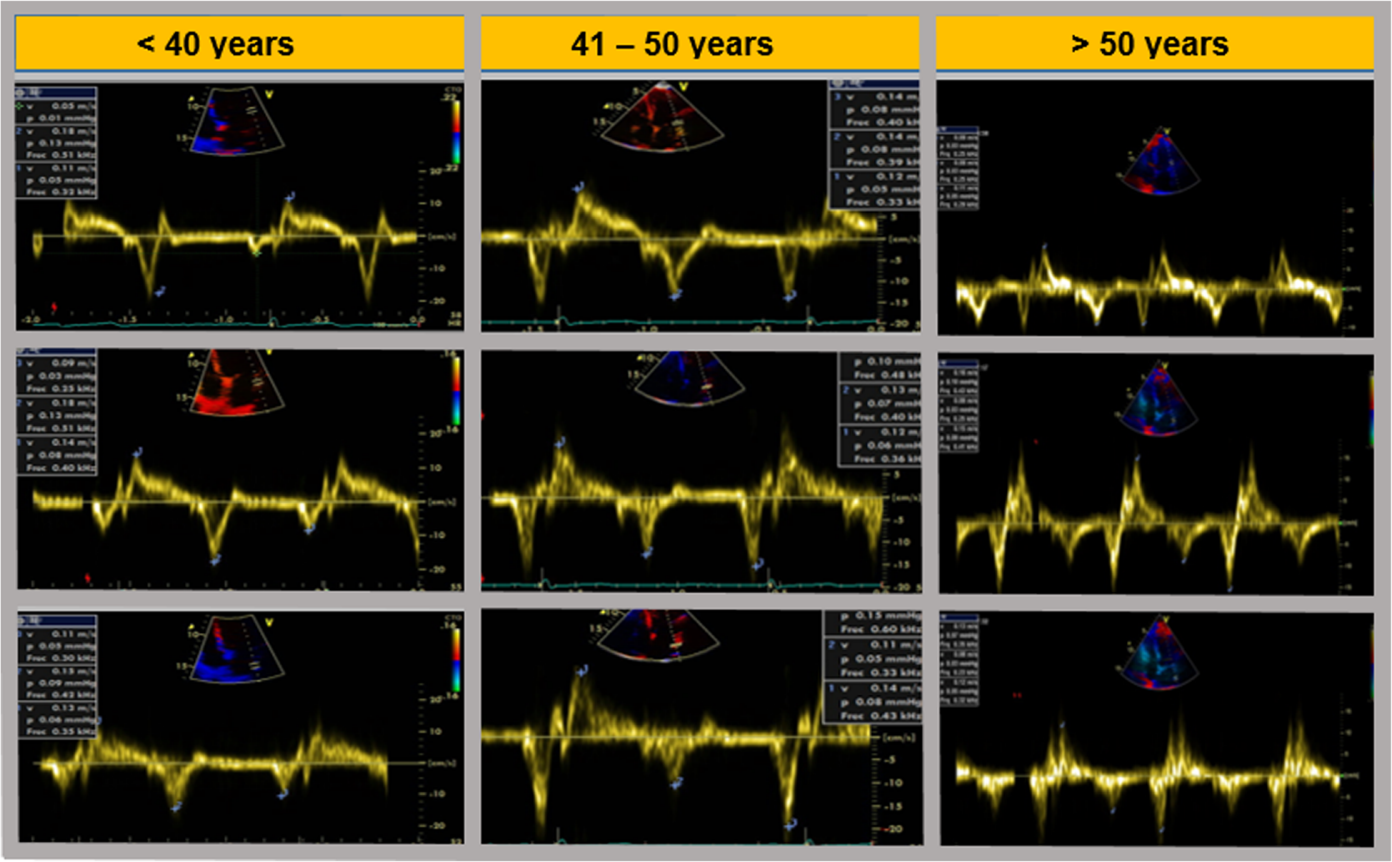
Pulsed TDI at the annular level (upper row), basal LA (middle row), and mid LA (lower row), according to age groups. TDI: Tissue Doppler Imaging, LA: left atrial

As shown in Fig. 4, in the present study, when comparing the results obtained at the annular and auricular levels by TDI, we observed that LA lateral wall has a different speed profile from the mitral annulus; while E’*la*/A’*la* ratio turned < 1 in the age-group older than 40 years old, Lma E´/Lma A´ ratio changed in the sixth decade, which suggests that by directly assessing the atrial myocardium we can detect changes that are not evident at the annulus level.

Inaba et al. evaluated the LA by SRI in a subgroup of healthy patients; they could not find a significant correlation of late diastole with age, which according to the author could be due to the quality of images and having analyzed only one of the walls [30]. Boyd et al. also evaluated a population of healthy adults with atrial SRI and reported significant correlation of SRI with age at the different phases: reservoir, conduit and atrial systole [1]. Similar findings were reported by Sun et al.; this author analyzed the ratio between early and late diastole (SRE/SRA) finding a negative correlation with age. These authors also demonstrated changes in atrial SRI that would allow earlier detection of impaired diastolic function with this method compare to other parameters usually used [34].

In a recent work Yoshida et al. evaluated the impact of age on cardiac function by speckle tracking SRI echocardiography and serum B type natriuretic peptide and concluded that the decrease in the reservoir and conduit phases are the earliest markers of age-related cardiac remodeling [35]. Regarding the atrial contraction function and its changes with age, the information is still controversial; some studies showed an increase in this function [20, 24] while others did not find significant changes [28, 33, 36].

In this study both TDI and SRI showed a modification in the velocities of the LA lateral wall in the age subgroups over 40 years old, at least a decade before the other methods analyzed.

Regarding reproducibility, Thorstensen et al. evaluated inter-observer variability of Lma S´ and Lma E´ by pulsed wave TDI and reported inter-observer COR of 2.1 cm/s and 5.5 cm/s, respectively, which are comparable to those observed in this study for S’*la* basal and E’*la* basal (COR 3.12 cm/s and COR 3.7 cm/s). The COR of Lma A´ was not reported in that study; instead, they reported a COR of 2.1 cm/s for the average value of mitral annulus A´ measured at the septal, lateral, inferior and anterior walls, which is inferior to the A’*la* basal COR of 3.82 cm/s observed in this study, as expected, since the authors reported that averaging measures of multiple walls resulted in a reduction of the error [37].

Thomas et al., who evaluated the LA lateral wall by TDI offline, reported an intra-observer mean difference of 0·06 cm/s with 95% limits of agreement between 0·42 and - 0·3 cm/s, and inter-observer mean difference of 0·07 cm/s with 95% limits of agreement between 0·61 and - 0·47 cm/s for A’*la* values measured at mid segments [33]. As the Hunt study showed, assessing variability by comparing repeated offline analyzes of the same recording previously acquired results in smaller values ​​of variability of each parameter analyzed. For instance, the authors described that inter and intra-analyzer average mean error for the global diastolic measurements was 44 and 64% lower than the average mean error calculated by separate recordings and analyses. This highlights the influence of the image acquisition process on intra e inter-observer variability, and could be a possible explanation for the differences observed between this study and that of Thomas et al.

#### Limitations

The main disadvantage of the TDI by spectral pulse online is that measurements are dependent on the insonation angle and can subject to translational movement of adjacent structures [38–41]. To overcome these limitations, in this study the measurements were made trying to maintain the narrowest possible angle of the segments to be measured, with a small sample volume and in expiratory apnea. We also evaluated and compared the results obtained at the basal and mid segments, and the average between basal and mid segments when both could be satisfactorily measured, observing similar results, which we believe would minimize the measurement error determined by the cardiac movement. When evaluating only one of the walls, we did not analyze the global functionality of the LA that can be modified by the other structures not included in this study. However, it proved to be useful to detect impaired diastolic function alike SRI, which we believe is nonetheless valuable.

In our experience, the basal segment of the lateral wall of the LA can be correctly aligned in almost all of the studied subjects, while the mid segments presented, in some cases, a difficult alignment to generate an adequate curve.

There could be a limitation to apply this method in structural disease such as mitral annular calcification or atrial fibrillation.

The size of the sample was limited and the population sample used for this study has not been selected by random methods, so the informed results must be confirmed with other studies.

## Conclusions

The effects of age on TDI online by spectral pulse of the LA lateral wall were assessed and normal values according to age group were reported. Age correlated with numerous parameters analyzed. Age-related changes in LA and diastolic function could be detected as early with this technique as with some parameters evaluated by SRI. Future studies are required to explore if this method could be used in low-income places to address in part, LA and diastolic function abnormalities in other populations with established cardiovascular disease or at risk of presenting it.

## Supplementary information

**Additional file 1: Supplemental Table 1.** Analysis of the variables by age subgroups.

**Additional file 2: Supplemental Table 2.** Correlations with age at mid and average segments with TDI and SRI.

**Additional file 3: Supplemental Table 3.** Univariate and multivariate analysis.

## Data Availability

The data and materials used in this study are available from the corresponding author on reasonable request.

## Abbreviations

AF: Atrial fibrillation
Av.: Average
BSA: Body surface area
COR: Coefficient of repeatability
DBP: Diastolic blood pressure
EF: Ejection fraction
HR: Heart rate
DTE: Deceleration time to peak E
*MD*: *Mean difference*
LA: Left atrial
LA Vol Ind: Left atrial volume index
IQR: Interquartile range
Lma: Lateral mitral annulus
LV: Left ventricular
Md: Median
SBP: Systolic blood pressure
*SD*: *Standard deviation*
Sma: Septal mitral annulus
TDI: Tissue Doppler Imaging
SRI: Strain Rate Imaging
Y: Years
