# Supplementary material for: Tissue Doppler Imaging and strain rate of the left atrial lateral wall: age related variations and comparison with parameters of diastolic function
Source: Cardiovasc Ultrasound. 2020 Sep 10;18:38. doi: 10.1186/s12947-020-00221-2 (PMC7488512; doi:10.1186/s12947-020-00221-2)
Supplement: Supplementary file 1 — Additional file 1: Supplemental Table 1. Analysis of the variables by age subgroups. [file 12947_2020_221_MOESM1_ESM.docx]

**Supplemental Table 1:** Analysis of the variables by age subgroups

|  | **Global** | **≤ 30 y**  **N: 14**  **Md (IQR)** | **31 - 40 y**  **N: 27**  **Md (IQR)** | **41 - 50 y**  **N: 27**  **Md (IQR)** | **51 - 60 y**  **N:14**  **Md (IQR)** | **≥ 61 y**  **N: 9**  **Md (IQR)** | **P** |
| --- | --- | --- | --- | --- | --- | --- | --- |
| ***S´la* mid (cm/s)** | 11 (3) | 11 (3) | 11 (3) | 11.5 (2) | 10 (3.7) | 12.5 (2.5) | NS |
| ***S´la* av. (cm/s)** | 11.5 (3) | 11.25 (3) | 11 (1.5) | 11.5 (2.3) | 10.5 (3.8) | 11.5 (1.5) | NS |
| ***E´la* mid (cm/s)** | 12 (5.2) | 15 (3.5) | 14 (3) | 12 (3.75) | 9.5 (1.7) | 8.5 (2) | <0.0001 |
| ***E´la* av. (cm/s)** | 12 (5.2) | 15.5 (3.6) | 14.5 (2.5) | 12.5 (2.3) | 9.75 (1.8) | 8.25 (1.2) | <0.0001 |
| ***A´la* mid (cm/s)** | 11 (4) | 10 (1.5) | 11 (3) | 12 (3) | 11 (1) | 14 (5.5) | 0.017 |
| ***A´la* av. (cm/s)** | 12 (3.2) | 10.25 (2.3) | 11 (2) | 12.5 (4) | 12.25 (2.8) | 15.25 (4.7) | 0.002 |
| ***E´la/A´la* mid** | 0.96(0.5) | 1.54 (0.7) | 1.27 (0.4) | 0.86 (0.4) | 0.78 (0.21) | 0.52 (0.1) | <0.0001 |
| ***E´la/A´la* av.** | 0.98(0..5) | 1.38 (0.4) | 1.31 (0.3) | 0.9 (0.3) | 0.77 (0.1) | 0.57 (0.1) | <0.0001 |
| **SRS mid (1/s)** | 2.24 (1) | 3 (0.8) | 2.56 (0.8) | 2.2 (0.6) | 1.87 (0.3) | 1.87 (0.4) | 0.003 |
| **SRS av. (1/s)** | 2.51 (0.8) | 3.31 (0.7) | 2.68 (0.7) | 2.33 (1.02) | 2.3 (0.7) | 1.73 (0.6) | 0.001 |
| **SRE mid (1/s)** | -2.04 (0.9) | -2.92 (1) | -2.32 (0.8) | -1.72 (0.8) | -1.92 (0.4) | -2.07 (0.5) | 0.0005 |
| **SRE av. (1/s)** | - 2.32(0.8) | -3.07 (0.9) | -2.71 (0.7) | -2.11 (1) | -2.12 (0.2) | -2 (0.3) | <0.0001 |
| **SRA mid (1/s)** | -2.25 (1) | -2.4 (1.4) | -2.1 (0.7) | -2.3 (0.9) | -2.26 (0.6) | -2.38 (1.8) | NS |
| **SRA av. (1/s)** | - 2.41 (1) | -2.45 (1.2) | -2.36 (0.8) | -2.44 (1) | -2.7 (0.5) | -2.48 (1.5) | NS |
| **SRE/SRA mid** | 0.93 (0.6) | 1.19 (0.5) | 1.18 (0.7) | 0.77 (0.4) | 0.78 (0.2) | 0.55 (0.3) | 0.029 |
| **SRE/SRA av.** | 0.94 (0.5) | 1.21 (0.3) | 1.15 (0.6) | 0.79 (0.7) | 0.78 (0.1) | 0.56 (0.3) | 0.005 |

*la*: left atrial, av.: average, SR: Strain Rate
