# Supplementary material for: Tissue Doppler Imaging and strain rate of the left atrial lateral wall: age related variations and comparison with parameters of diastolic function
Source: Cardiovasc Ultrasound. 2020 Sep 10;18:38. doi: 10.1186/s12947-020-00221-2 (PMC7488512; doi:10.1186/s12947-020-00221-2)
Supplement: Supplementary file 2 — Additional file 2: Supplemental Table 2. Correlations with age at mid and average segments with TDI and SRI. [file 12947_2020_221_MOESM2_ESM.docx]

**Supplemental Table 2:** Correlations with age at mid and average segments with TDI and SRI

| Parameter | R | P |
| --- | --- | --- |
| *S´la* mid (cm/s) | 0 | NS |
| *S´la* average (cm/s) | - 0.04 | NS |
| *E´la* mid (cm/s) | - 0.59 | < 0.0001 |
| *E´la* average (cm/s) | - 0.66 | < 0.0001 |
| *A´la* mid (cm/s) | 0.34 | NS |
| *A´la* average. (cm/s) | 0.39 | NS |
| *E´la/A´la* mid | - 0.65 | < 0.0001 |
| *E´la/A´la* average | - 0.73 | < 0.0001 |
| SRS mid (1/s) | - 0.46 | 0.03 |
| SRS average (1/s) | - 0.48 | 0.01 |
| SRE mid (1/s) | 0.49 | 0.009 |
| SRE average (1/s) | 0.61 | < 0.0001 |
| SRA mid (1/s) | - 0.04 | NS |
| SRA average (1/s) | - 0.06 | NS |
| SRE/SRA mid | - 0.39 | NS |
| SRE/SRA average | - 0.47 | 0.02 |

la: left atrial, SR: strain Rate, TDI: Tissue Doppler Imaging, SRI: Strain Rate Image
