# Supplementary material for: Tissue Doppler Imaging and strain rate of the left atrial lateral wall: age related variations and comparison with parameters of diastolic function
Source: Cardiovasc Ultrasound. 2020 Sep 10;18:38. doi: 10.1186/s12947-020-00221-2 (PMC7488512; doi:10.1186/s12947-020-00221-2)
Supplement: Supplementary file 3 — Additional file 3: Supplemental Table 3. Univariate and multivariate analysis. [file 12947_2020_221_MOESM3_ESM.docx]

**Supplemental Table 3:** Univariate and multivariate analysis

| **Log *S´la* basal** | **Simple linear regression** | | | **Multiple linear regression** | | |
| --- | --- | --- | --- | --- | --- | --- |
|  | **B** | **p** | **R2** | **B** | **p** | **R2 model / p model** |
| **Age**  **Female**  **SBP**  **DBP**  **HR**  **BSA** | -0.0006917  -0.07911  0.006690  0.0044310.005822 0.1820 | 0.7  0.0987  0.01380.01210.00873 0.11 | --  --  0.08101  0.07819  0.07816  -- | --  --  0.002574  0.001592  0.005868  -- | --  --  0.30074  0.68072  0.00885  -- | R2 0.1721; p 0.003668 |
| **Log *E´la* basal** | **Simple linear regression** | | | **Multiple linear regression** | | |
|  | **B** | **p** | **R2** | **B** | **p** | **R2 model; p model** |
| **Age**  **Female**  **SBP**  **DBP**  **HR**  **BSA** | -0.014088  -0.05995  -0.005124  -0.007387  -0.0003044  0.004915 | 2.65e-12  0.331  0.035  0.0484  0.916  0.973 | 0.4283  --  0.0579  0.05094  --  -- | -0.014146  --  0.001918  -0.002552  --  -- | 2.97e-09  --  0.510  0.553  --  -- | R2 0.4234; p 8.526e-09 |
| **Log *A´la* basal** | **Simple linear regression** | | | **Multiple linear regression** | | |
|  | **B** | **p** | **R2** | **B** | **p** | **R2 model; p model** |
| **Age**  **Female**  **SBP**  **DBP**  **HR**  **BSA** | 0.006939  0.007908  0.009342  0.011704  0.002772  0.06225 | 0.000328  0.883  1.49e-06  0.000136  0.276  0.626 | 0.1371  --  0.2671  0.1775  --  -- | 0.005466  --  0.006055  0.002170  --  -- | 0.00512  --  0.02363  0.57699  --  -- | R2 0.3457; p 7.852e-07 |
| **Log (*E´la/A´la*) basal** | **Simple linear regression** | | | **Multiple linear regression** | | |
|  | **B** | **p** | **R2** | **B** | **p** | **R2 model; p model** |
| **Age**  **Female**  **SPB**  **DBP**  **HR**  **BSA** | -0.021335  -0.05493  -0.014809  -0.019345  -0.003202  -0.08982 | 5.97e-16  0.514  2.50e-06  8.11e-05  0.425  0.646 | 0.5225  --  0.2544  0.1859  --  -- | -0.019839  --  -0.004460  -0.004242  --  -- | 1.00e-12  --  0.168  0.370  --  -- | R2 0.6304; p 5.65e-16 |

SBP: systolic blood pressure, DBP: diastolic blood pressure, HR: heart rate, BSA: body surface area
